# Occurrence of Aflatoxin M_1_ (AFM_1_) in Donkey Milk Collected in Northern Italy

**DOI:** 10.3390/vetsci7040176

**Published:** 2020-11-12

**Authors:** Alberto Altafini, Marco Tassinari, Alessandro Guerrini, Paola Roncada

**Affiliations:** Department of Veterinary Medical Sciences, University of Bologna, Via Tolara di Sopra 50, 40064 Ozzano dell’Emilia (Bologna), Italy; alberto.altafini@unibo.it (A.A.); marco.tassinari@unibo.it (M.T.); alessandro.guerrini5@unibo.it (A.G.)

**Keywords:** aflatoxin M_1_, donkey milk, food safety, HPLC-FLD, immunoaffinity columns

## Abstract

Aflatoxin M_1_ (AFM_1_) is a well-known mycotoxin that can be found in the milk of animals that have ingested feed contaminated with aflatoxin B_1_ (AFB_1_). In Italy, the development of donkey farms is mainly due to growing request of donkey milk, which is considered an incomparable substitute for human mother’s milk for its chemical composition and organoleptic characteristics. The aim of this study was to assess the occurrence of AFM_1_ in donkey milk produced in a farm in Northern Italy, also in view of the few data available about the presence of this mycotoxin in this type of milk. Therefore, 63 milk samples were collected and analyzed using a fast and sensitive HPLC and fluorescence detection (FLD) method previously optimized and validated. None of the milk samples collected were found to be contaminated at a level above the limit of quantification (LOQ) (0.0125 ng/mL), while only one sample showed traces of the mycotoxin at a concentration between the limit of detection (LOD) and LOQ (0.0044 ng/mL), well below the legal limit established for infant milk and follow-on milk (0.025 ng/mL). These results are in line with those of the few similar surveys carried out on donkey milk and seem to indicate a low risk of AFM_1_ contamination for this food.

## 1. Introduction

Donkey’s milk is considered the most similar to mother’s milk for its chemical composition and organoleptic characteristics. For this reason, it has become a staple food in the diet of infants affected by cow’s milk protein allergy (CMPA). The incidence of this disease of infancy and early childhood seems to be prevalent in the developed countries, where it ranges from 0.5 to 3% in the first year of life [1]. Caseins are one of the main allergenic components of milk. Donkey milk has a casein concentration lower than cow’s milk, and it is characterized by a greater digestibility, clinical tolerability, palatability, and nutritional adequacy. Clinical studies showed that donkeys’ milk is well tolerated (82.6–88%) by infants [2]. Furthermore, it can provide additional physiological functions, such as antibacterial substances, digestive activity molecules, growth factors, and hormones [3,4]. For its properties, several authors envisage a role of this food in the osteogenesis process, in treatment of atherosclerosis, in the post-heart-attack recovery period, in cases of premature senescence, and in hypocholesterolemic diets [5]. Moreover, the use of donkey milk in external-dermal application for cosmetic and medical purposes is well known, and its beneficial effects are due to milk proteins, which contain all essential amino acids, which may help restore the protein layer of cell membranes and intracellular compartments of cells [6].

The donkey (*Equus asinus*) is a rustic, undemanding, and easily adaptable animal, which in most cases lends itself to semi-wild breeding, and is able to enhance marginal areas [7]. In the 1950s, in Italy, there were almost a million donkeys, but their numbers have decimated over time, and it is only since 2000 that the number of donkeys started increasing again, saving many local breeds from extinction. Indeed, since 2007, their numbers have increased by 90% in 10 years reaching 59,000 heads. The biggest boost to the development of donkey farms come from the growing request of donkey milk due to fact that in Italy around 15,000 children every year are born with gastrointestinal allergies due to cow’s milk intolerance [8].

Aflatoxins (AFs) are the mycotoxins of the greatest concern to food safety due to their wide distribution in foods and feeds and their high toxicities [9]. AFs are mainly produced by the *Aspergillus* species, in particular *A. flavus*, *A. parasiticus*, and *A. nominus*, and these fungi may invade agricultural products intended for human and animal consumption, such as maize grains, cereals, nuts, peanuts, and other oil fruits [10,11], as well as hays and ensiled forages [12]. Among the 400 known mycotoxins, aflatoxins B_1_ (AFB_1_), B_2_ (AFB_2_), G_1_ (AFG_1_), and G_2_ (AFG_2_) are the most significant mycotoxins in foods and feeds [13,14,15]. Aflatoxin M_1_ (AFM_1_) is a hydroxylated product of AFB_1_ in humans and animals, which may be present in milk from animals fed with aflatoxin B_1_ contaminated feed [16,17,18,19]. The main AFB_1_ metabolism pathways depend on different CYP450 isozymes. The metabolic conversions include the epoxidation to highly toxic AFB_1_-8,9-epoxide (AFBO), ketoreduction to moderately toxic aflatoxicol (AFL), hydroxylation to mildly toxic AFM_1_ and relatively nontoxic AFB_2a_ or AFQ_1_, and demethylation to a relatively nontoxic AFP_1_ [20,21]. In ruminants, AFs are only partly degraded by the ruminal flora, and a typical secondary metabolite of rumen metabolism is aflatoxicol [22,23]. The remaining fraction is absorbed in the digestive tract by passive diffusion and is hydroxylated in the liver to aflatoxin M_1_ [10,24,25]. AFM_1_ can be conjugated to glucuronic acid and excreted via bile or enters the systemic circulation from where it can be excreted in the urine or in milk [23,26]. Moreover, the in vitro biotransformation of AFB_1_ to AFM_1_ has been shown experimentally in bovine mammary epithelial cells [27]. AFM_1_ is primarily considered a detoxification product of AFB_1_ metabolism, showing only 10% of mutagenicity and carcinogenic potency compared to its precursor [25,28], although its acute toxicity is nearly equal to that of AFB_1_ [29,30]. The International Agency for Research on Cancer (IARC) classified AFs as a group as carcinogenic to humans (Group 1) causing hepatocellular carcinomas (HCCs) [31]. 

Signs of aflatoxin exposure in mammalians are decreased appetite, decrease in growth rate, lowered milk production and reduced feed efficiency, icterus, liver damage, immunosuppression, and anemia [32,33]. In dairy cattle, the symptoms of acute aflatoxicosis include anorexia, depression, dramatic drop in milk production, weight loss, lethargy, gastrointestinal dysfunctions such as ascitis, icterus, tenesmus, abdominal pain, bloody diarrhea, decreased feed intake and efficiency, weight loss, jaundice, abortion, hepatoencephalopathy, blindness, walking in circles, ear twitching, frothy mouth, photosensitization, bleeding, and death [34]. In equines, the signs of aflatoxicosis reported are jaundice, depression, anorexia, lameness, and death [35].

AFM_1_ is not a metabolite exclusive of mammals, and in fact, it is produced by microsomal liver preparations from many non-mammalian species [29]. Thus, for example, several studies report the presence of this mycotoxin in poultry. AFM_1_ was detected in chicken organs (liver, gizzard, and heart) collected from different retail markets in Egypt [36]. AFM_1_ residues were measured in the livers of broiler chicks in an experiment to evaluate the effectiveness of an adsorbent to remove AFs from contaminated feed [37]. Jurišić et al. found AFM_1_ in excreta and ileal content of broilers receiving a diet containing AFs [38]. Small quantities of AFM_1_ have also been reported in eggs [39].

In view of the hazardous and widespread nature of these types of contaminants, the European Community has set maximum levels in foodstuffs for human consumption and products intended for animal feed. In particular, for AFB_1_ in feed materials, a maximum content of 0.02 mg/kg was established, while in complementary and complete feed, the limit was set at 0.01 mg/kg [40]. For AFM_1_, the maximum tolerated level in raw milk, heat-treated milk, and milk for the manufacture of milk-based products was set at 0.05 µg/kg, while a more restrictive limit (0.025 µg/kg) was established for infant milk and follow-on milk [41]. Under particular climatic conditions, especially in terms of temperature and humidity, the development of aflatoxigenic molds in cereals and other crops can occur, and in such cases, subsequent controls in milk very often show levels of AFM_1_ higher than normal as a result of ingestion of contaminated feed by milk-producing animals. Similar conditions occurred in Italy in 2003 [30,42] and in 2012 [43,44].

The aim of the present study was to assess the occurrence and levels of AFM_1_ in donkey milk produced in a farm of the North of Italy, also in view of the few data available about the presence of this mycotoxin in this particular type of milk. For the analysis, a sample preparation procedure with immunoaffinity columns (IAC) was used, and a fast and sensitive HPLC method coupled with fluorescence detection (FLD) was optimized and validated. 

## 2. Materials and Methods

### 2.1. Animals, Diets, and Nutrition

The milk came from donkeys of the following breeds: Martina Franca, Amiata, Sant’Andrea, San Domenico, Argentato di Sologno, Sardo, Ragusano, Poitou. The animals were raised outdoors free to move in large spaces within a rural area. The feeding was mainly the spontaneous vegetation coming during grazing integrated with feed. The composition of the feed was as follows: barley, flaked barley, wheat bran, flaked corn, flaked fava beans, flaked soybean meal, dehydrated alfalfa flour, sugar beet pulp, sugar cane molasses, fatty acids (fat of vegetable origin), dicalcium phosphate, calcium carbonate from ground calcium rocks, sodium chloride, and brewer’s yeast.

### 2.2. Sampling

A total of 63 milk samples were collected between June and November 2015 from a farm located in Northern Italy. The sampling was carried out mainly in autumn (52 samples collected) and, to a lesser extent, in summer (11 samples collected). Each milk sample was from a single milking of an animal, except for one sample, which was collected from the farm bulk milk tank. Milk samples were stored in 50 mL Falcon tubes at −20 °C until analysis, without first having been subjected to any type of treatment.

### 2.3. Solvents and Reagents

The reference standard of AFM_1_ was purchased from Sigma-Aldrich Co. (St Louis, MO, USA). Water, acetonitrile, and methyl alcohol used to prepare AFM_1_ standard solutions, for the sample clean-up procedure, and to formulate the HPLC mobile phase, were HPLC grade. All solvents were obtained from Mallinckrodt Baker B.V. (Deventer, The Netherlands). Immunoaffinity columns used for samples purification (Afla M_1_^TM^ HPLC) were purchased from Vicam^®^ (Milford, MA, USA). 

### 2.4. Chromatographic Apparatus and Conditions

AFM_1_ analysis was performed by HPLC-FLD on a chromatographic apparatus consisting of a System Gold Programmable Solvent Module 126 Pump (Beckman, San Ramon, CA, USA) equipped with an HT 800 L autosampler (HTA, Brescia, Italy) fitted with a 100 µL loop. Chromatographic separation was achieved in isocratic elution mode and at room temperature using an analytical column Zorbax Eclipse Plus C18 150 × 4.6 mm 5 µm (Agilent, Santa Clara, CA, USA). The mobile phase consisted of 61% of deionized water, and 39% of acetonitrile. The flow rate was set at 0.7 mL/min, and the injection volume was 100 µL. The FLD detection was obtained by means of a 821 FP fluorescence detector (Jasco, Tokyo, Japan) set at the excitation wavelength of 360 nm and emission wavelength of 440 nm. The system was computer-controlled by a Beckman Coulter 32 Karat Software.

### 2.5. Sample Preparation and Immunoaffinity Clean-Up Procedures

The procedure was set up on the basis of the instructions provided by the immunoaffinity column manufacturer with a few modifications. Forty milliliters of milk were measured into a 50 mL cylinder, transferred into a 50 mL Falcon tube, and centrifuged in a refrigerated centrifuge at 5 °C for 15 min (1540× *g*). The fat layer was then removed and the skim portion, once at ambient temperature, was loaded onto a IAC (Afla M_1_^TM^ HPLC) and passed at a flow rate of about 1–2 drops per second. After washing the column two times with 10 mL of deionized water, AFM_1_ was eluted in a glass tube with 1.25 mL of acetonitrile-methyl alcohol (3:2) and 1.25 mL of deionized water at a rate of 1 drop per 2–3 s. The eluate collected was reduced to dryness in a rotational vacuum concentrator (Univapo Martinsried/Munich, Germany). The residue was redissolved in 1 mL of acetonitrile-methyl alcohol (3:2 *v*/*v*) and diluted with 1 mL of deionized water. Finally, the resulting solution was filtered through a 0.45 μm nylon syringe filter (Phenomenex, Torrance, CA, USA) and transferred to a HPLC vial for analysis (AFM_1_, if present in milk sample, will become concentrated 20 times in the final analysis solution).

### 2.6. Quantification

A commercial AFM_1_ standard solution 10 µg/mL in acetonitrile (Sigma-Aldrich Co., St. Louis, MO, USA) was diluted to give a stock solution 1 µg/mL and subsequently divided into 1 mL aliquots, which were stored at −20 °C until use. Working solutions, obtained by diluting an aliquot of stock solution with water, were used to prepare reference standard in water–acetonitrile–methanol solution (5:3:2 *v*/*v*) at 6 concentration levels (0.25, 0.5, 1, 1.5, 2, 2.5 ng/mL), and these were analyzed by HPLC-FLD to generate reference curves in solvent. Spiked samples were prepared by adding appropriate volume of AFM_1_ working solution to blank samples of milk and then processed as for the unknown samples. The resulting solutions were analyzed by HPLC-FLD to generate calibration curves in matrix. The quantities of AFM_1_ to be added to milk samples were calculated so that the concentrations of the final solutions were the same of the reference standards in solvent (0.25, 0.5, 1, 1.5, 2, 2.5 ng/mL) and correspond to concentrations of 0.0125, 0.025, 0.05, 0.075, 0.1, and 0.125 ng/mL in matrix, taking into account the concentration factor resulting from the sample extraction/purification procedure (20:1). Calibration curves were obtained from least squares linear regression analysis and were used to determine AFM_1_ concentrations in the unknown samples. Regression equations and determination coefficient (R^2^) were calculated using Microsoft Excel 2013 software.

An in-house validation of the analytical method was carried out and included the following parameters: selectivity, calibration curve performance (including linearity and working range), limit of detection (LOD) and quantification (LOQ), accuracy, precision, recovery, and carry-over. Selectivity was proved using 15 blank samples, which were analyzed and evaluated for interference. Linearity was determined in the working range on the basis of the determination coefficient (R^2^). Working range was the range of values from the LOQ value to the maximum concentration of AFM_1_ in the standard solution for which the calibration curve had been plotted. LOQ was the lowest calibration standard of the 6-point calibration curve. LOD was calculated as the concentration corresponding to a signal 3 times the noise level measured in 15 blank samples at the AFM_1_ retention time. The signal–concentration relationship was determined based on the lowest calibration standard (LOQ). Accuracy and precision were assessed on samples spiked with known amounts of AFM_1_ and were measured at 4 different concentrations over 4 consecutive days (16 determinations in total). To evaluate accuracy, bias to nominal concentration was calculated, while coefficient of variation (CV) of the replicate measurements was taken as a measure of precision. The calculation of recovery was made by dividing peak areas of extracted standard solutions from AFM_1_-spiked samples by peak areas of AFM_1_ standard solutions at the same concentration. Carry-over was assessed by injecting blank samples after the highest calibration standard. Quality control (QC) samples were processed and analyzed together with the unknown samples to monitor the performance of the extraction–purification procedure and to assess the validity of the analytical results. This study was performed according to ISO 9001 requirements.

## 3. Results and Discussion

### 3.1. Method Development

Compared to the extraction/purification procedure suggested by the IAC manufacturer, a smaller volume of milk was used for each sample (40 mL vs. 50 mL). The final eluate collected from IACs, instead of being analyzed directly, was reduced to dryness and subsequently redissolved (1 mL of acetonitrile–methanol solution and 1 mL of water) to obtain a more accurate measure of the volume of final solution (2 mL), and to maintain a concentration factor 20:1 in relation to the smaller amount of milk loaded onto the IAC. This is an important step of the clean-up procedure, because an inaccurate final elution can result in considerable errors in the evaluation of the concentration of the analyte. The chromatographic condition were developed starting from a method already tested in our laboratory for this type of analysis. Several parameters were optimized to reduce the run time and improve the sensitivity of the method. For the initial tests, a conventional analytical column Luna C18 250 × 4.6 mm 5 µm (Phenomenex, Torrance, CA, USA) was used, and the mobile phase composition was 70% of water-methanol (90:10 *v*/*v*) and 30% of acetonitrile, in isocratic elution. The flow rate was set at 0.9 mL/min, and the injection volume was 100 µL. At these conditions, the retention time of AFM_1_ was 6.5 min and the total run time was 9 min. Since the samples were quite clean and the analyte to be quantified was only one, it was decided to test a shorter column (150 mm). Firstly a column Zorbax Eclipse plus C18 4.6 × 150 mm 3.5 µm (Agilent, Santa Clara, CA, USA) was used, and under the same conditions (flow rate, mobile phase composition, and injection volume), peak height increased by 8% and retention time decreased by 35%. Analyses using smaller injection volumes (50 and 75 µL) were also carried out, but despite the improvement of some chromatographic parameters (peak width and retention time decreased, peak shape increased), peak height decreased without a reduction in the signal-to-noise ratio. A series of experiments using linear gradients of acetonitrile from 30 to 44% with different slopes were performed, but no significant improvements were observed, also considering the re-equilibration time requested in the gradient elutions, while isocratic elution with 65% water and 35% acetonitrile seemed to lead to satisfactory analyses. However, after several injections of extracted samples, the backpressure of the column increased too much for a conventional HPLC system such as the apparatus used in this study. Consequently, the column was replaced with a similar one packed with 5 µm particles. After several runs using both linear gradients of acetonitrile from 30 to 40% and isocratic elutions with percentages of acetonitrile in the same range, the best result was obtained by applying an isocratic elution with mobile phase consisting of 61% water and 39% acetonitrile at a flow rate of 0.7 mL/min. In respect to the initial analytical conditions, the use of a shorter column together with the optimization of the chromatographic parameters has resulted in a significant reduction in AFM_1_ retention time (from 6.5 to 3.8 min) and of the total analysis time (from 9 to 5 min), as well as in an improvement of the detection limit as a consequence of the narrowing of the peak width and increase in peak height.

### 3.2. Assay Validation

**Figure 1:**
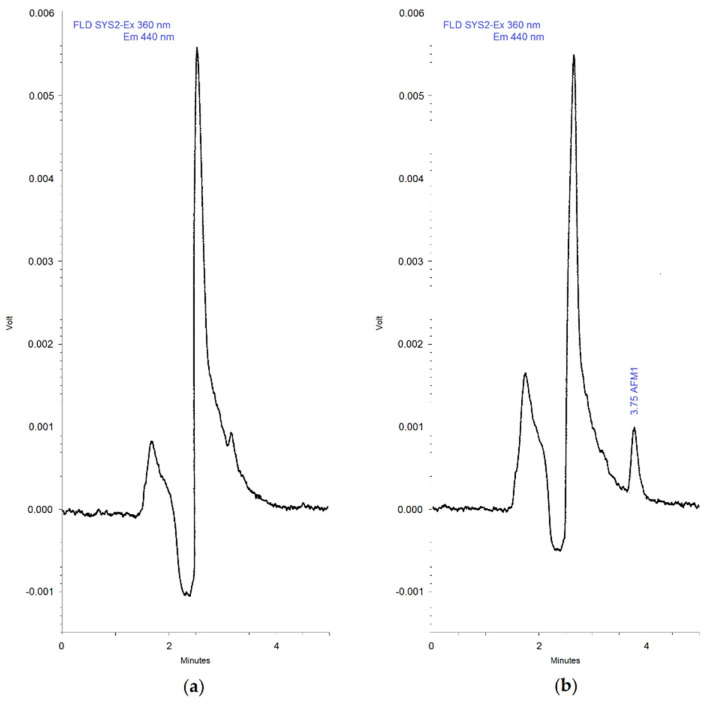
Chromatograms of a blank sample of milk (**a**) and a blank sample of milk spiked with Aflatoxin M_1_ (AFM_1_) at 0.025 µg/L level (**b**).

The 15 blank samples extracted and analyzed for the evaluation of selectivity did not show interfering peaks at the retention time window of AFM_1_, as well as no abnormal background noises being observed. The retention time was 3.7 min while the run time was 5 min. Figure 1 shows representative chromatograms of a blank sample of milk (a) and a blank sample of milk spiked with AFM_1_ at 0.025 µg/L level (b). Both reference curves in solvent generated from analysis of pure AFM_1_ standard solutions and calibration curves in matrix, obtained from analysis of standard solutions resulting from extraction of spiked milk samples, showed a satisfactory linearity in the range of concentrations tested, which went from 0.0125 to 0.125 ng/mL (working range). In fact, in all regressions, the coefficient of determination (R^2^) was always >0.999. LOQ and LOD of the method were 0.0125 and 0.0025 ng/mL, respectively, well below the AFM_1_ limits established at EU level for milk (0.05 ng/mL), infant milk, and follow-on milk (0.025 ng/mL). Accuracy and precision were determined by preparing and analyzing four samples per level at four concentration levels (LOQ, low, medium, and high) covering the working range, and the concentrations tested were 0.0125, 0.025, 0.075, 0.125 ng/mL. The bias ranged from −6 to −2.5%, while the coefficient of variation (CV) was in the range 5.1–12.8%. Recovery experiments were carried out at four spike levels within the working range, and the average recovery percentages were found to be between 86.6 and 92.2%, while the overall average recovery was 89.0%. The data about the percent recoveries and the mean recoveries for each fortification level are shown in Table 1. Injection of blank samples following the highest calibration standard did not show peaks above LOD, thus proving that no significant carry-over effects occurred. Therefore, the overall results of the method validation demonstrated the reliability of the method for the determination of AFM_1_ in milk. 

The extraction–purification technique based on the use of immunoaffinity columns together with HPLC-FLD analysis is still a valid approach for this type of determination especially when more sophisticated and expensive equipment such as HPLC-MS/MS are not available. The refinement of the method carried out in this study has allowed a general improvement of several analytical parameters that have led to lower consumption in the mobile phase, reduction in analysis time, and therefore, reduction in costs.

### 3.3. Occurrence of AFM_1_ in Milk Samples

In the present survey, 63 samples of donkey milk were analyzed, and none of them were found to be contaminated with AFM_1_ at a level above the LOQ, while only one sample showed traces of the mycotoxin at a concentration between LOD and LOQ (0.0044 ng/mL). Milk samples and AFM_1_ concentrations are reported in Table 2.

In the evaluation of the results, the law limit established for infant milk and follow-on milk (0.025 ng/mL) was kept as a reference, as donkey milk is mainly used as infant food. The only sample in which AFM_1_ was detected had a concentration much lower than the abovementioned limit. These data prove that the milk analyzed is safe for human consumption, at least regarding the presence of this toxin, and suitable for young children. Others similar surveys carried out in Italy but on ruminants’ milk, generally showed a higher percentage of positive samples [26,42,45,46,47,48,49,50,51]. Whilst considering the limited number of samples analyzed and their provenience from one single farm, some hypothesis can be formulated to explain the findings of the present study. The donkeys from which the milk was taken are kept for most of the year in the pasture and therefore the only way for the animals to take AFs is through the feed given in the evening when they returns to the stables. Given the almost total absence of the toxin in milk, it can be assumed that the feed was safe and effective controls have always been carried out. Another explanation could be that the non-detection of AFM_1_ in practically all milk samples is due to the very low casein content of donkey milk compared to the cow milk [52,53,54,55]. In fact, this toxin has a high affinity for casein [50,56,57,58,59], and therefore in the milk of animals exposed to AFs via feed, there could be a correlation between the amount of AFM_1_ and the percentage of casein. A further possibility is that the virtual absence of AFM_1_ is due to a low carry-over of AFM_1_ in the milk of donkey. While for lactating cows a large amount of literature exists, in the case of lactating donkeys, there is very little information on the transfer of AFM_1_ into milk. The only reference study is the one reported by Tozzi et al., which evaluated the amount of AFM_1_ and AFM_2_ in donkey milk in response to feeding AFB_1_ and AFB_2_. For 12 days, six donkeys received a diet supplemented with naturally contaminated corn containing the two toxins (202µg of AFB_1_ and 11 µg of AFB_2_). The maximum levels detected in milk were 32.25 ng/L (AFM_1_) and 23.33 ng/L (AFM_2_), and carry-over into milk resulted to be 0.02% and 0.31% for AFM_1_ and AFM_2_, respectively [12]. By focusing on AFM_1_, the percentage of carry-over is very low as compared with that reported in other species.

Several experiments were carried out using dairy cows fed AFB_1_ contaminated diets, and the ratio between the AFM_1_ in milk and the intake of AFB_1_ was calculated. Pettersson et al., in a study in which six cows were fed AFB_1_ contaminated diets at two levels (5.3 and 10.8 µg/kg), in a change-over experiment to groups of three animals for one week, reported that the overall mean carry-over was 2.54 ± 0.76% [60]. Masoero et al. investigated the relationship between somatic cell counts, milk yield, and conversion of dietary AFB_1_ into milk AFM_1_ using 34 lactating dairy cows. The AFB_1_ carry-over into milk calculated that the plateau was between 1.29 and 2.70%, and resulted to be affected by the milk yield [61]. In a study carried out on 18 dairy cows to evaluate the efficacy of a mycotoxin deactivating product, Pietri et al. reported that the carry-over of AFB_1_ in milk was 3.85% in animals who ingested a daily dose of AFB_1_ equal to 97.3 µg for seven days [62]. Sumantri et al. reported a carry-over of AFB_1_ in milk equal to 0.1% in dairy cows who ingested AFB_1_ at low and high doses (3.4 and 350 µg AFB_1_/cow/day) for 10 days [63]. In another experiment carried out by Britzi et al., 12 cows were used to determine AFM_1_ carry-over following daily administration of feed containing 86 μg AFB_1_ for seven days. The mean carry-over rate at steady-state was 5.8% and 2.5% in mid-lactation and late-lactation groups, respectively [64]. Gonçalves et al. used 20 Holstein cows in late lactation to evaluate the effect of different sources of *Saccharomyces cerevisiae* on AFM_1_ excretion in milk from cows receiving 480 µg AFB_1_ per day for six days. In the control group (animals who ingested AFB_1_ contaminated feed without *Saccharomyces-cerevisiae*-based products), the carry-over rate was in the range 0.83–3.89% [65]. In an experiment to quantify AFM_1_ carry-over rate from feed to cheese, Costamagna et al. reported that in dairy cattle receiving an average daily dose of 66.8 µg AFB_1_ via natural contaminated feed, the carry-over to milk was 0.84% [58].

The abovementioned experiments show that, in dairy cows, AFB_1_ carry-over to milk ranged from 0.1 to 5.8%. These studies also show that factors influencing carry-over include milk yield, feed consumption, and the ratio of concentrated feed included in the diet, as reported also by Völkel et al. [66]. Furthermore, carry-over rate can vary widely among individual animals, from day to day and from one milking to the next, as it is influenced by various (patho-)physiological factors [67].

Fewer similar studies were carried out on ruminants of other species. Battacone et al. reported two experiments carried out on dairy ewes. In the first one, four animals received a single dose of AFB_1_ (2 mg), and the mean rate of transfer of AFB_1_ into AFM_1_ in milk was 0.032%. In the second experiment, 12 ewes divided into three groups received different daily doses of AFB_1_ (32, 64, and 128 μg) for 14 days, and AFB_1_ carry-over in milk was 0.082, 0.133, and 0.119% for each group, respectively [68]. In a later similar study carried out using 12 ewes divided into three groups receiving 32, 64, and 128 μg AFB_1_ per day for seven days, higher carry-over values were found (0.33, 0.29, 0.26% for each experimental group, respectively) [69].

Scientific literature reports that these type of evaluations were carried out also on goats. In an experiment in which three goats received a single dose of AFB_1_ (0.8 mg), Mazzette et al. found that the mean rate of transfer of AFB_1_ into AFM_1_ in milk was 0.26%, with a high individual variability [70]. In a very similar study carried out by Battacone et al., five dairy goats were administered 0.8 mg of AFB_1_ orally, and about 0.17% of the amount of the toxin administered was detected as AFM_1_ in milk [71]. Nageswara Rao et al. used nine lactating goats in early lactation to evaluate the effect of two inert adsorbents on AFM_1_ excretion in milk from animals receiving AFB_1_ at the rate of 100 µg/kg in the diet for 14 days. In the control group (animals who ingested AFB_1_ contaminated feed without adsorbents), the carry-over rate measured after three and 14 days was 0.140% ± 0.049 and 0.397% ± 0.124, respectively [72]. Similar results were obtained in an almost identical experiment carried out by Mugerwa et al. In the goats of the control group, the carry-over rate measured after three and 14 days was 0.11 and 0.30%, respectively [73].

The surveys on the presence of AFM_1_ in donkey milk are really very few and in many cases, the number of samples analyzed is low. The findings of such studies show no or few positive samples and low concentrations of mycotoxin, as reported in Table 3.

With respect to the table above, in the survey carried out in Serbia in 2013, the authors suggest that the high contamination frequency observed could be consequence of the hot and dry weather conditions of 2012 that lead to the development of aflatoxigenic molds in maize and feed [74]. The study carried out in Croatia in 2013 does not report the percentage of positive samples, but it specifies that AFM_1_ levels exceeded the LOQ value (23.2 ng/L) in only 59 samples of cow milk and two samples of goat milk. The authors state that these positive samples were linked to the use of contaminated supplementary feedstuff [75]. The survey carried out in Sicily from 2013 to 2016 reports the complete absence of AFM_1_ in donkey milk, and that such milk comes from animals that lived freely [57]. Since the feed of grazing animals consists mainly of grass and hay, which are not major sources of intake for toxins, the possibility that these animals assume AFs through the feed is very low. The findings of the survey carried out in Greece in 2015 are in line with those of the studies mentioned above (few positive samples and low concentrations detected). According to the authors, these data can be due to the type of feed (the animals were reared on pasture), and to the very low carry-over of aflatoxin B_1_ to M_1_ that has been reported for donkeys [76]. Finally, also in the case of the study carried out in Germany, the authors suggest that the absence of AFM_1_ in the milk samples can be explained by the feeding of the animals that consisted of grass and hay, supplemented by pelleted feed and oat grains, which were tested negative for AFB_1_ [77].

## 4. Conclusions

In conclusion, the data of the present study together with those of the other surveys carried out on donkey milk seem to be reassuring about the safety of this type of milk. However, as the main consumers of this food are infants and young children, it is worth stressing the importance of regularly monitoring donkey milk, although it is a niche product, including checks on the feed administered to the animals in order to ensure its healthiness. The present study is only a survey focusing on one set of samples from one single farm. Considering the few data on the mycotoxin contamination of this type of milk (to date only one survey carried out in Italy), it would be interesting to continue the research extending it to the milk from other donkey farms to have a more complete picture of the AFM_1_ risk in this food.

## Figures and Tables

**Table 1:** Recovery data of the method for analysis of AFM_1_ in samples of donkey milk spiked at four concentration levels.

AFM_1_ Spiking Levels (µg/L)	Recovery (%) ^1^	M (%) ^2^
0.0125	87.7	89.0
0.025	86.6	
0.075	89.7	
0.125	92.2	

^1^ Number of replicates: 4. ^2^ Average recoveries of the 4 spiking levels.

**Table 2:** Milk samples and AFM_1_ concentrations.

Milk Sample	Animal	Sampling Period	AFM_1_ (µg/L)	Milk Sample	Animal	Sampling Period	AFM_1_ (µg/L)
1	41	Jun–Aug	<LOD	33	96	Nov	<LOD
2	33	Jun–Aug	<LOD	34	22	Nov	<LOD
3	64	Jun–Aug	<LOD	35	25	Nov	<LOD
4	41	Jun–Aug	<LOD	36	41	Nov	<LOD
5	13	Jun–Aug	<LOD	37	83	Nov	<LOD
6	83	Jun–Aug	<LOD	38	45	Nov	<LOD
7	6	Jun–Aug	<LOD	39	90	Nov	<LOD
8	23	Jun–Aug	<LOD	40	86	Nov	<LOD
9	92	Jun–Aug	<LOD	41	97	Nov	<LOD
10	6	Jun–Aug	<LOD	42	13	Nov	<LOD
11	23	Jun–Aug	<LOD	43	93	Nov	<LOD
12	92	Nov	<LOD	44	60	Nov	<LOD
13	94	Nov	<LOD	45	92	Nov	<LOD
14	89	Nov	<LOD	46	36	Nov	0.0044
15	22	Nov	<LOD	47	91	Nov	<LOD
16	25	Nov	<LOD	48	65	Nov	<LOD
17	41	Nov	<LOD	49	9	Nov	<LOD
18	83	Nov	<LOD	50	85	Nov	<LOD
19	45	Nov	<LOD	51	23	Nov	<LOD
20	90	Nov	<LOD	52	94	Nov	<LOD
21	13	Nov	<LOD	53	22	Nov	<LOD
22	29	Nov	<LOD	54	25	Nov	<LOD
23	64	Nov	<LOD	55	41	Nov	<LOD
24	58	Nov	<LOD	56	83	Nov	<LOD
25	42	Nov	<LOD	57	45	Nov	<LOD
26	23	Nov	<LOD	58	90	Nov	<LOD
27	66	Nov	<LOD	59	86	Nov	<LOD
28	94	Nov	<LOD	60	97	Nov	<LOD
29	21	Nov	<LOD	61	13	Nov	<LOD
30	54	Nov	<LOD	62	60	Nov	<LOD
31	89	Nov	<LOD	63 ^1^	-	Nov	<LOD
32	1	Nov	<LOD				

^1^ Sample collected from the farm bulk milk tank.

**Table 3:** Results of surveys carried out on donkey milk and other types of milk to monitor AFM_1_ occurrence.

Sampling Area	Sampling Period	Type of Milk	N° Samples Collected	% Positive	Concentration Range		References
					(µg/kg o ng/L)		
					Min	Max	
Serbia	2013	Donkey milk	5	60	0.005 ^1^	0.035 ^1^	[74]
		Cow milk	150	98.7	0.01 ^1^	1.2 ^1^	
		Goat milk	10	80	0.008 ^1^	0.24 ^1^	
		Breast milk	10	60	0.006 ^1^	0.022 ^1^	
		Infant formula	1	0	-	-	
Croatia	2013	Donkey milk	14	− ^3^	3.43 ^2^	10.4 ^2^	[75]
		Cow milk	337	− ^3^	2.69 ^2^	162.3 ^2^	
		Goat milk	32	− ^3^	2.78 ^2^	40.8 ^2^	
		Sheep milk	19	− ^3^	2.11 ^2^	5.87 ^2^	
Sicily (Southern Italy)	2013–2016	Donkey milk	84	0	-	-	[57]
		Cow milk	170	11.1	− ^3^	0.03 ^1^	
		Sheep milk	133	3.6	− ^3^	0.15 ^1^	
Greece	2015	Donkey milk	36	13.9	5.6 ^2^	26.5 ^2^	[76]
Germany	− ^3^	Donkey milk	6	0	-	-	[77]

^1^ µg/kg. ^2^ ng/L. ^3^ Not mentioned in the references.
